# Molecular cytogenetic characteristics of new spring bread wheat introgressive lines resistant to stem rust

**DOI:** 10.18699/vjgb-24-43

**Published:** 2024-07

**Authors:** O.A. Baranova, I.G. Adonina, S.N. Sibikeev

**Affiliations:** All-Russian Institute of Plant Protection, St. Petersburg-Pushkin, Russia; Institute of Cytology and Genetics of the Siberian Branch of the Russian Academy of Sciences, Novosibirsk, Russia; Federal Center of Agricultural Research of the South-East Region, Saratov, Russia

**Keywords:** Triticum aestivum L., introgressive wheat lines, alien introgressions, Puccinia graminis f. sp. tritici, Ug99, Sr genes, Triticum aestivum L., интрогрессивные линии пшеницы, чужеродные интрогрессии, Puccinia graminis f. sp. tritici, Ug99, Sr гены

## Abstract

Anticipatory wheat breeding for pathogen resistance is key to preventing economically significant crop losses caused by diseases. Recently, the harmfulness of a dangerous wheat disease, stem rust, caused by Puccinia graminis f. sp. tritici, was increased in the main grain-producing regions of the Russian Federation. At the same time, importation of the Ug99 race (TTKSK) is still a possibility. In this regard, the transfer of effective resistance genes from related species to the bread wheat breeding material followed by the chromosomal localization of the introgressions and a marker analysis to identify known resistance genes is of great importance. In this work, a comprehensive analysis of ten spring bread wheat introgressive lines of the Federal Center of Agricultural Research of the South-East Region (L657, L664, L758, L935, L960, L968, L971, L995/1, L997 and L1110) was carried out. These lines were obtained with the participation of Triticum dicoccum, T. timopheevii, T. kiharae, Aegilops speltoides, Agropyron elongatum and Secale cereale. In this study, the lines were evaluated for resistance to the Ug99 race (TTKSK) in the Njoro, Kenya. Evaluation of introgression lines in the field for resistance to the Ug99 race (TTKSK) showed that four lines were immune, two were resistant, three were moderately resistant, and one had an intermediate type of response to infection. By cytogenetic analysis of these lines using fluorescent (FISH) and genomic (GISH) in situ hybridization, introgressions from Ae. speltoides (line L664), T. timopheevii (lines L758, L971, L995/1, L997 and L1110), Thinopyrum ponticum = Ag. elongatum (2n = 70) (L664, L758, L960, L971, L997 and L1110), as well as introgressions from T. dicoccum (L657 and L664), T. kiharae (L960) and S. cereale (L935 and L968) were detected. Molecular markers recommended for marker-oriented breeding were used to identify known resistance genes (Sr2, Sr25, Sr32, Sr1A.1R, Sr36, Sr38, Sr39 and Sr47). The Sr36 and Sr25 genes were observed in lines L997 and L1110, while line L664 had the Sr39+Sr47+Sr25 gene combination. In lines L935 and L968 with 3R(3D) substitution from S. cereale, gene resistance was presumably identified as SrSatu. Thus, highly resistant to both local populations of P. graminis and the Ug99 race, bread wheat lines are promising donors for the production of new varieties resistant to stem rust.

## Abstract (ru)

Опережающая селекция пшеницы на устойчивость к патогенам – залог предотвращения экономически значимых потерь урожая от болезней. В последние годы в основных зернопроизводящих областях Российской Федерации наблюдается увеличение вредоносности опасного заболевания пшеницы – стеблевой ржавчины (возбудитель Puccinia graminis f. sp. tritici). В то же время сохраняется опасность заноса на территорию России расы патогена Ug99 (TTKSK), которая угрожает производству зерна во всем мире. В связи с этим большое значение приобретают перенос эффективных генов резистентности от родственных видов в селекционный материал мягкой пшеницы, выявление хромосомной локализации интрогрессий и проведение маркерного анализа для идентификации известных генов устойчивости. В настоящей работе был проведен комплексный анализ десяти интрогрессивных линий яровой мягкой пшеницы селекции Федерального аграрного научного центра Юго-Востока (Л657, Л664, Л758, Л935, Л960, Л968, Л971, Л995/1, Л997 и Л1110), полученных с участием Triticum dicoccum, T. timopheevii, T. kiharae, Aegilops speltoides, Agropyron elongatum и Secale cereale. Оценка интрогрессивных линий в полевых условиях на устойчивость к расе Ug99 (TTKSK) показала, что четыре линии были иммунны, две – устойчивы, три – среднеустойчивы, а одна имела промежуточный тип реакции на заражение. Цитогенетический анализ с помощью методов флуоресцентной (FISH) и геномной (GISH) гибридизации in situ выявил интрогрессии от Ae. speltoides (линия Л664), T. timopheevii (линии Л758, Л971, Л995/1, Л997 и Л1110), Thinopyrum ponticum = Ag. elongatum (2n = 70) (Л664, Л758, Л960, Л971, Л997 и Л1110), а также интрогрессии от T. dicoccum (Л657 и Л664), T. kiharae (Л960) и S. cerealе (Л935 и Л968). Для идентификации известных генов устойчивости (Sr2, Sr25, Sr32, Sr1A.1R, Sr36, Sr38, Sr39 и Sr47) использовали молекулярные маркеры, рекомендованные для маркер-ориентированной селекции. Наличие генов Sr36 и Sr25 было постулировано у двух линий (Л997 и Л1110), генов Sr39, Sr25 и Sr47 – у линии Л664. У линий Л935 и Л968 c замещением 3D(3R) от S. cereale ген устойчивости к стеблевой ржавчине предположительно определен как SrSatu. Высокоустойчивые как к местным популяциям P. graminis, так и к расе Ug99 линии мягкой пшеницы являются перспективными донорами для создания новых устойчивых к стеблевой ржавчине сортов.

## Introduction

One of the conditions for increasing the yield of bread wheat is the production of varieties that are resistant to biotic and abiotic stressors. The set of the most harmful biostressors for bread wheat includes a group of rust disease pathogens: Puccinia triticina f. sp. tritici Erikss., P. striiformis f. sp. tritici Erikss., P. graminis f. sp. tritici Erikss. & Henning. These pathogens cause epiphytoties of brown, yellow and stem rust. The harmfulness of each of them can reach 50 % (Knott, 1989). The causative agents of these diseases are characterized by high virulence and great diversity in racial composition (Gultyaeva et al., 2021, 2022; Baranova et al., 2023b).

In the global production of bread wheat and under Russian conditions, a special place is occupied by stem rust (pathogen P. graminis f. sp. tritici (Pgt)), which can cause yield losses of more than 80 % during epiphytotic development on susceptible varieties. The well-known race of stem rust pathogen Ug99 (TTKSK) and its variants, which infect wheat varieties and lines with effective resistance genes Sr31, Sr36 and Sr24, still pose a real threat to wheat production in the regions of the African continent, the Middle East and Asia. Due to the possibility of fungal spores spreading with air masses over vast distances, a threat of the pathogen being introduced into the territory of Eurasian countries, including Russia, remains. Over the last decade, in Europe, Kazakhstan, China and the Russian Federation, aggressive races of the fungus have appeared that are not related to the Ug99 race, but have caused severe outbreaks of the disease (Vasilova et al., 2017; Lewis et al., 2018; Baranova et al., 2021; Patpour et al., 2022).

Low diversity of stem rust resistance genes is a common problem in commercial wheat varieties around the world. The adult resistance gene Sr57 (Lr34/Yr18/Pm38/Bdv1), which is part of a locus with pleiotropic action that determines nonspecific resistance to biotrophic pathogens, as well as juvenile resistance genes such as Sr38, Sr6Agi, Sr25 and Sr31 are used in Russian domestic varieties. The Sr31 gene still remains effective against stem rust in the Russian Federation (Baranova et al., 2023b). Genes Sr6Agi and Sr25 lose effectiveness in the Volga region, but are effective against Western Siberian populations of the fungus (Kelbin et al., 2020; Baranova et al., 2021). The Sr38 gene is ineffective against Volga populations of the pathogen, but is recommended for breeding in Western Siberia (Skolotneva et al., 2021).

To expand the genetic basis of varieties, it is extremely important to obtain breeding material diverse for resistance genes. In general, this problem is solved by involving related species of bread wheat, mainly from the secondary and tertiary gene pools. Currently, 26 out of 63 stem rust resistance genes have been transferred from the genomes of related species (McIntosh et al., 2013, 2022). The Ae. speltoides, T. timopheevii, T. dicoccum, T. ponticum, S. cereale species remain important sources of valuable genes for resistance to fungal diseases and in particular to stem rust for practical breeding of bread wheat (McIntosh et al., 2013). Genes Sr32, Sr39, Sr47 were transferred from Aegilops speltoides (Taush) (SS, 2n = 14) to the wheat genome; Sr36, Sr37, Sr40, from Triticum timopheevii Zhuk. (AtAt GG, 2n = 28); Sr31, Sr27, Sr1A.1R, Sr50, from Secale cereale L. (RR, 2n = 14) (McIntosh et al., 2013). Effectiveness against P. graminis and the nature and size of the introgressed material are important aspects of using these genes to develop resistant bread wheat varieties. It is important to produce combinations of currently effective Sr genes with each other or with genes that have partially lost their effectiveness, or with adult resistance genes.

At the Federal Center of Agricultural Research of the South-East Region (FCAR of the South-East Region), work is underway to produce new breeding material using relatives of bread wheat. Previously, lines produced with the participation of a wide range of species showed high resistance to leaf rust in the conditions of the Saratov Volga region (Gultyaeva et al., 2020). The aim of our work is a comprehensive study of new introgressive lines including assessment of resistance to the Ug99 (TTKSK) stem rust race, chromosomal localization of alien introgressions and identification of Sr genes using molecular markers

## Materials and methods

Plant material. Ten introgressive lines of spring bread wheat from FCAR of the South-East Region were studied. Their pedigree, indicating the donor of alien genetic material, is given in Table 1.

**Table 1.**
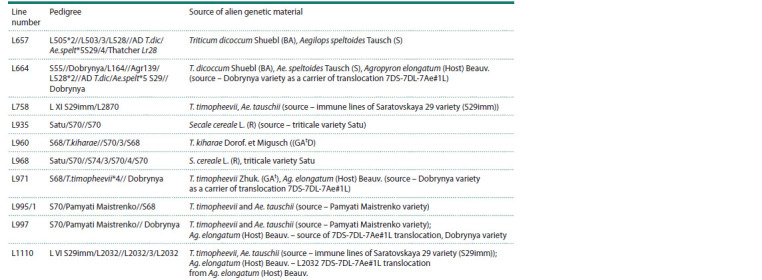
Pedigree of spring bread wheat introgressive lines Notе. The pedigree lines indicate the following varieties of spring bread wheat: L503, L505, Dobrynya, Saratovskaya 29 (S29), Saratovskaya 55 (S55), Saratovskaya 68 (S68), Saratovskaya 70 (S70), Saratovskaya 74 (S74), as well as lines L164, L528, L2870, L2032, Agr139, L VI S29 imm, L XI S29 imm of spring bread wheat.

Cytogenetic analysis. Preparations of mitotic chromosomes were prepared from the meristem of seedling roots in accordance with the method (Badaeva et al., 2017). The FISH (fluorescence in situ hybridization) method using probes based on various repetitive sequences: Spelt1 (Salina et al., 1997) and Spelt52 (Salina et al., 2004), pSc119.2 (Bedbrook et al., 1980) and apAs1 (Rayburn, Gill, 1986) was used to analyze the karyotype of the lines. The FISH method described in the work of Salina et al. (Salina et al., 2006) with minor modifications was used. GISH (genomic in situ hybridization) using labeled S. cereale genomic DNA as a probe was performed according to previously published work (Schubert et al., 1998). The preparations were analyzed using an Axio Imager M1 microscope (Zeiss, Germany) equipped with a ProgRes MF CCD digital camera and Isis software (Meta Systems, Germany).

Phytopathological analysis. Resistance to race Ug99 (TTKSK) analysis was carried out at the adult plant stage using a modified Cobb scale (Peterson et al., 1948) in 2023 at the plant pathology nurseries at the International Maize and Wheat Improvement Center (CIMMYT) at the Kenya Agricultural and Livestock Research Organization (KALRO) in Njoro. The main distinguishing feature of the Ug99 race pathotypes is virulence towards carriers of the Sr31 gene. The degree of damage to varieties with the Sr31 gene in KALRO plant pathology nurseries in the growing season of 2023 was: for the variety Prokhorovka (Sr31) – 60 % (60MSS), for the variety Yugo-Vostochnaya 2 (Sr31) – 80 % (80S), for the variety Saratovskaya 74 (without Sr genes) – 80 % (80S).

Molecular genetic analysis. DNA was isolated from fiveday- old wheat seedlings using cetyltrimethylammonium bromide (CTAB method) (Murray, Thompson, 1980). To identify the resistance genes Sr2, Sr32, Sr1A.1R, Sr36, Sr38, Sr39, Sr47, DNA markers recommended for marker-assisted selection (MAS) were used. A list of molecular markers used in the work with links to sources is presented in the Supplementary Material 11. PCR was performed in duplicate on a C1000 Thermal Cycler (manufactured by BioRad). Amplification products were separated on 2 % agarose and 8 % polyacrylamide gels stained with ethidium bromide. Isogenic lines and varieties with known Sr genes served as a positive control; the susceptible variety Khakasskaya served as a negative control. PCR mixture was taken without adding DNA to control contamination. GeneRulerTM 50bp DNA Ladder (Thermo Scientific) was used as a molecular weight marker. Visualization of amplification products was carried out using the ChemiDoc™ (Bio-Rad) gel documentation system.


Supplementary Materials are available in the online version of the paper: https://vavilov.elpub.ru/jour/manager/files/Suppl_Baranova_Engl_28_4.pdf


## Results

Phytopathological analysis of spring bread wheat introgressive lines

Phytopathological screening of the lines at the stage of adult plants showed that all lines were resistant to the Ug99 race to varying degrees: four lines were immune (infection type 0), two were resistant (R), three were moderately resistant (MR) to this highly aggressive race of the fungus (Table 2). The only exception was one line, L995/1, which had an intermediate infection type (M) with 5 % of disease development.

**Table 2.**
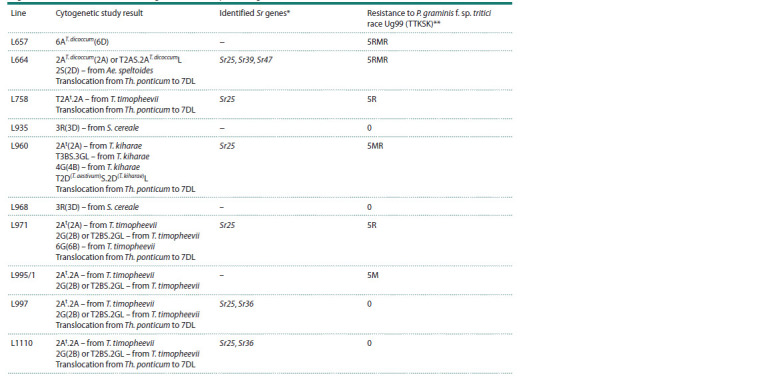
Characteristics of spring bread wheat introgressive lines by translocations/substitutions, Sr genes and resistance to stem rust (Ug99) at the adult plants stage * The genes, the identification of which was confirmed by cytogenetics and pedigree analysis, are presented; the Sr25 gene was identified previously (Baranova et al., 2023a). ** Resistance: 0 – immune infection type, R – resistant, MR – medium–resistant, RMR – intermediate type of infection between resistance and medium resistance, M – intermediate type of infection between medium resistance and medium susceptibility.

Cytogenetic analysis of spring bread wheat introgressive lines

The identification of alien genetic material and determination of its state in the reconstructed genome of bread wheat in the form of addition or substitution chromosomes and translocations were the aim of the introgression lines cytogenetic analysis

The main results of cytogenetic analysis are presented in Table 2 and in the Figure. Additional information indicating the probe combinations used is provided in the Supplementary Material 2.

**Fig. 1.**
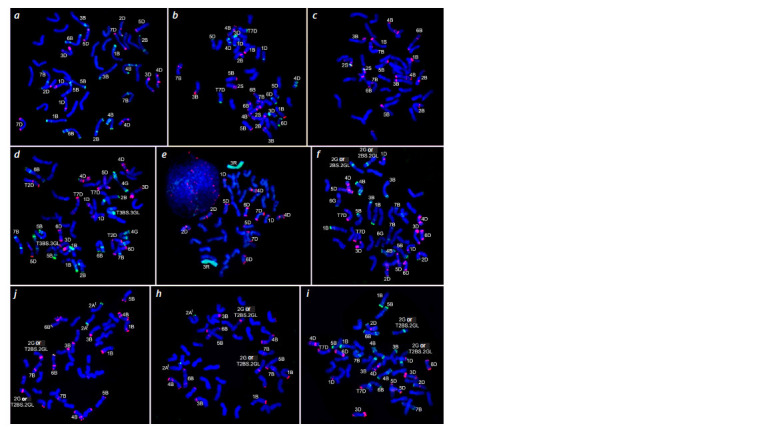
Results of FISH and GISH with different probe combinations on metaphase chromosomes of bread wheat introgressive lines. Probes pSc119.2 (green), pAs1 (red): a – line L657, b – L664, d – L960, f – L971, i – L1110; probes Spelt52 (green), pSc119.2 (red): c – L664, h – L997; probes Spelt1 (green), pSc119.2 (red): g – L997; Rye DNA (green), pAs1 (red): e – line L968.

Karyotyping of the lines showed that each of them is characterized by the standard number of chromosomes for hexaploid wheat – 42. FISH was performed with probes pSc119.2 and pAs1 for each of the ten lines. The probe pSc119.2 (Bedbrook et al., 1980) is predominantly localized on the chromosomes of the bread wheat genome B, and pAs1 (Rayburn, Gill, 1986) is predominantly localized on the chromosomes of the D genome. The simultaneous use of these probes allows the identification of all chromosomes of the B and D genomes and some chromosomes of the A genome (Schneider et al., 2003). In addition, it is possible to identify the chromosomes of the G genome of T. timopheevii by the localization of hybridization signals with the pSc119.2 probe (Jiang, Gill, 1994). GISH with S. cereale DNA was used to analyze two wheat lines that had rye among ancestors. Analysis of eight lines, the ancestors of which included Ae. speltoides, T. timopheevii or T. kiharae, involved hybridization with probes Spelt1 and Spelt52 (performing GISH with DNA from these species is difficult due to their close relationship to bread wheat).

Lines L657 and L664 were obtained with the participation of Ae. speltoides. In the L657 line, Spelt52 repeat sites were not identified, and the Spelt1 probe is localized at the ends of the chromosome 6B arms. According to previous studies, this localization of Spelt1 occurs in bread wheat varieties (Salina et al., 2006). Consequently, we cannot speak with confidence about translocations from Ae. speltoides in this lineage. FISH with the pAs1 probe showed the absence of 2D chromosomes in the L664 line and revealed a pair of chromosomes with a weak signal of pSc119.2 on the short arm and two signals on the long arm (see the Figure, b), which also contains the Spelt52 site (see the Figure, c). We identified this chromosome as chromosome 2S of Ae. speltoides (Badaeva et al., 1996; Ruban, Badaeva, 2018). Thus, in the case of line L664, we have established chromosomal substitution 2S(2D). In addition, the results of hybridization of probes pSc119.2 and pAs1 on the chromosomes of the L657 line (see the Figure, a) indicate the substitution of chromosome 6D, presumably with chromosome 6A of T. dicoccum, a species that is present in the pedigree of this line. In the L664 line, Spelt1 sites were identified at the long arm ends of the genome A chromosomes pair, most likely chromosomes 2A. In bread wheat varieties, such localization of this probe has not been observed, but it is characteristic of tetraploid wheats, in particular T. dicoccum, and may indicate chromosomal substitution or translocation from a given species (present in the pedigree).

Lines L758, L960, L971, L995/1, L997, and L1110 were expected to have introgressions from the species T. timopheevii or T. kiharae. Interestingly, weak hybridization signals with the Spelt52 probe were detected on the short arms of the genome A chromosome pair, most likely chromosome 2A in all six lines (see the Figure, h). This localization of Spelt52 is characteristic of T. timopheevii or T. kiharae and may indicate translocations from these species. No hybridization signals with the Spelt1 probe were detected in the L758 line. Lines L995/1, L997 and L1110 carry Spelt1 blocks at the ends of the short arms of chromosome 6B (see the Figure, g), which is typical for a number of bread wheat varieties (Salina et al., 2006). The localization of Spelt1 on the long arms of chromosome 2A in the lines L960, L971, L997 and L1110 (see the Figure, g) in combination with the localization of the Spelt52 probe on the short arms of these chromosomes (see the Figure, h) may indicate the substitution of chromosome 2A with 2At (from T. timopheevii or T. kiharae, respectively) in these lines. In line L960, another Spelt1 site is located on the long arm of the chromosome, which, according to the localization of the pSc119.2 probe, corresponds to chromosome 4G of T. timopheevii, while chromosome 4B is absent (see the Figure, d ). The results obtained indicate that the L960 line has chromosomal substitution 4G(4B). Also, based on the localization of probe pSc119.2 in the L960 line, translocation T3BS.3GL can be assumed. The distribution of the pAs1 probe on the long arm of chromosome 2D in this line is almost identical to that in T. kiharae, which indicates a probable translocation T2D(T. aestivum)S.2D(T. kiharae)L (see the Figure, d ). It should be noted that in the L960 line, as well as in the L758, L664, L971, L997 and L1110 lines, the localization of the pAs1 probe on the long arm of chromosome 7D does not correspond to bread wheat, which indicates a translocation (see the Figure, b, f, i). The presence of the Sr25 gene in four of these lines (Baranova et al., 2023a), transferred to the bread wheat genome from Th. ponticum (Friebe et al., 1996), and the hybridization pattern of pAs1 on the long arm of wheatgrass chromosome Js-7 (Cui et al., 2018), allow us to conclude that chromosome 7D of these lines carries translocations from Th. ponticum.

In the case of the L995/1, L971 and L997, L1110 lines, according to the results of hybridization with the pSc119.2 probe, we can talk about the substitution of chromosome 2B with 2G T. timopheevii, 2G(2B), or about the translocation of T2BS.2GL (see the Figure, f–i). Additionally, the L971 line is expected to have a chromosomal substitution 6G(6B) (see the Figure, f ).

Lines L935 and L968 were obtained using the Australian triticale variety Satu. GISH with rye DNA and FISH with probes pSc119.2 + pAs1 showed the substitution of 3D chromosomes with a pair of 3R chromosomes in these lines (see the Figure, e).

Identification of stem rust resistance genes using molecular markers

The results of Sr genes identification in the analyzed lines using molecular markers, confirmed by pedigree analysis and cytogenetic analysis data, are presented in Table 2. In this work, PCR fragments specific for genes Sr32, Sr39, Sr47 (Ae. speltoides), Sr36 (T. timopheevii) and Sr38 (Ae. ventricosa) were found in different lines. All the obtained results of PCR analysis, indicating the molecular markers used, are given in the Supplementary Material 3. The diagnostic fragment of the VENTRIUP-LN2 marker for the Sr38 gene was observed only in the L971 line (Supplementary Material 3). The presence of the Sr36 gene was established in two lines, L997 and L1110, using the Xstm773-2 marker (Table 2, Supplementary Material 3).

The Sr39 gene was identified using the Sr39#22 marker. A diagnostic fragment (800 bp) was detected in five lines (Supplementary Materials 3 and 4). To identify the Sr32 gene, the csSr32#2 marker was used. The diagnostic fragment was observed in three lines: L960, L968 and L995/1. The Sr47 gene was identified using three markers – Xgwm501, Xgpw4043, and Xgwm47 (Supplementary Materials 3 and 5). The diagnostic fragment of the Xgwm501 marker (109 bp) was identified in four lines: L971, L995/1, L997 and L1110. Only the 95-bp fragment of the two diagnostic fragments of the Xgpw4043 marker was amplified in the L657, L664, L758, and L971 lines; the 115-bp fragment was absent (Supplementary Material 5). The diagnostic fragment of the Xgwm47 marker (165 bp) was identified only in line L664.

Previously, all the lines we analyzed were tested for the presence of the Sr25 gene (Baranova et al., 2023a) using the Gb marker recommended for marker-based selection (Prins et al., 2001). This gene was identified in six lines (Table 2, Supplementary Material 3). According to the results of previous studies (Baranova et al., 2023a) and this work, the Sr2, Sr24, Sr28, Sr31, Sr1A.1R and Sr57 genes were not found in any of the lines

## Discussion

Efficiency of molecular markers recommended for marker-based selection for identifying stem rust resistance genes

Molecular markers are widely used to identify resistance genes to various pathogens including stem rust. Among the huge number of molecular markers, the most specific ones are highlighted and recommended for marker-based selection (https://maswheat.ucdavis.edu/). However, during work with a variety of plant material, especially with introgressive lines, a researcher may encounter insufficient specificity of even a recommended marker and, as a result, false-positive gene identification. In this regard, it is desirable to conduct complex studies and confirm the presence of the desired gene along with molecular genetic analysis data, study of pedigrees, cytogenetic and phytopathological results

During our work, introgression lines were analyzed cytogenetically and using molecular markers. Data from pedigree lines were also taken into account

In six out of the ten studied lines (L664, L758, L960, L971, L997 and L1110), the Sr25 gene was previously identified (Baranova et al., 2023a), which was fully confirmed by the cytogenetic analysis data in this work (Table 2). The Sr25 gene is linked to the leaf rust resistance gene Lr19 and is localized in the T7DS-7DL-7Ae#1L translocation from Th. ponticum (Friebe et al., 1994).

The identification of the Sr36 gene using the Xstm773-2 marker is also confirmed by cytogenetic analysis. As is known, the Sr36 gene is localized on chromosome 2G (Friebe et al., 1996). Lines L997 and L1110, in which this gene was identified according to molecular genetic analysis, carry chromosome 2G from T. timopheevii (see the Figure, g–i, Table 2).

We obtained ambiguous results regarding the resistance genes Sr32, Sr39 and Sr47, the source of which is Ae. speltoides. Cytogenetic analysis revealed genetic material from Ae. speltoides (substitution of chromosome 2D with chromosome 2S – 2S(2D) only in line L664, in the pedigree of which this species is present). However, the diagnostic fragment of the Sr39#22 marker (the Sr39 gene marker) was also identified in lines L971, L995/1, L997 and L1110 (Supplementary Material 3), which lack Ae. speltoides in their pedigrees but there is genetic material from T. timopheevii, which was confirmed cytogenetically. Also, based on the pedigrees of the L997 and L1110 lines, Dr. Savov’s synthetic (GAtD) was used in crosses (T. timopheevii × T. tauschii). Thus, the diagnostic fragment of the Sr39#22 marker was amplified in lines with material from T. timopheevii and possibly T. tauschii. It should be noted that similar results for the Sr39#22 marker were obtained by E.I. Gultyaeva and colleagues (Gultyaeva et al., 2014). Their study noted that, despite the fact that this marker is widely used to identify the Sr39/Lr35 gene, its diagnostic fragment was amplified in wheat samples with material from T. timopheevii and T. tauschii: for example, in the variety Pamyati Maistrenko, which was used to obtain lines L995/1 and L997 (Table 1).

The diagnostic fragment of the Sr32 gene marker csSr32#2 (152 bp) was identified in lines L960, L968 and L995/1 with genetic material from T. kiharae and Th. ponticum (line L960), S. cereale L. (line L968) and T. timopheevii (line L995/1) (Supplementary Material 3). Based on all of the above, we did not take into account the results obtained for this marker of the Sr32 gene, considering them a clear example of a falsepositive gene identification.

Another gene from Ae. speltoides is Sr47, which we identified using three markers: Xgwm501, Xgwm47 and Xgpw4043, the results were also ambiguous. The diagnostic fragment (109 bp) of the Xgwm501 marker was clearly detected in lines L971, L995/1, L997 and L1110 (Supplementary Material 3), which were described above. The Pamyati Maistrenko variety and Dr. Savov’s synthetic (GAtD) – T. timopheevii × T. tauschii are present in the pedigrees of those lines. As can be seen from cytogenetic analysis, the genetic material from Ae. speltoides is not present in them (Table 2). As for the Xgpw4043 marker, the diagnostic fragment of 95 bp was observed in lines L657, L664, L758 and L971, while the second diagnostic fragment of 115 bp was absent. This situation was described in the article (Klindworth et al., 2012), where in some wheat lines with the Sr47 gene the 115-bp fragment was not amplified or differed in staining intensity and only the 95-bp fragment was amplified and the authors strongly recommended to pick up several markers for Sr47 gene identification. The diagnostic fragment of the Xgwm 47 marker was identified only in the L664 line, which has Ae. speltoides in its pedigree, and cytogenetic analysis revealed chromosomal substitution 2S(2D). Also, in this line, diagnostic fragment of the Sr39#22 marker (the Sr39/Lr35 gene) was identified. It should be noted that both the Sr47 and Sr39 genes are localized on chromosome 2S of Ae. speltoides, with Sr39 on the short arm and Sr47 on the long arm (Klindworth et al., 2012). Since the L664 line has a 2S(2D) substitution, it is quite possible that it has both the Sr39 and Sr47 genes.

The Sr38 gene comes from the species Ae. ventricosa Tausch. and is linked to the genes for resistance to brown (Lr37) and yellow (Yr17) rust (Bariana, McIntosh, 1993). Despite the absence of this species in the pedigrees of the introgressive lines (Table 1), the diagnostic fragment of the VENTRIUP-LN2 marker to the Sr38 gene was present in the L971 line. Cytogenetic analysis did not reveal genetic material from Ae. ventricosa in this line, therefore we can conclude that in this case there was a false positive identification of the Sr38 gene

Thus, attention should be paid to the shown in our study insufficient specificity of the markers for the following genes: Sr32 – csSr32#2, Sr38 – VENTRIUP-LN2, Sr39 – Sr39#22, Sr47 – Xgwm501 and Xgpw4043. During identification of resistance genes using molecular markers in wheat samples with alien genetic material, this point must be taken into account. It is necessary to once again note the importance of combining different approaches when conducting the analysis of introgressive forms to increase its effectiveness

Characteristics of new spring bread wheat introgressive lines resistant to stem rust

The Supplementary Material 3 presents the results of assessing the resistance of the analyzed lines to the Volga region populations of the stem rust pathogen at the seedling stage, which we obtained earlier (Baranova et al., 2023a). It was shown that two lines (L657 and L971) were susceptible to the Tatarstan population of the fungus collected from the Nadira variety, while the L971 line was heterogeneous in resistance. Lines L758 and L960 were susceptible to the Saratov population collected from the Voevoda variety. Six lines showed resistance to both populations of the pathogen (L664, L935, L968, L995/1, L997, L1110). Thus, the characterization of introgressive lines will be based on the data from previous works (Baranova et al., 2023a, b) and the results obtained in this study.

All the lines were highly resistant to race Ug99 (TTKSK) with the exception of L995/1 as assessed by KALRO (Kenya) (Table 2). According to FAO data, to date the following genes remain effective versus the Ug99 race: Sr28, Sr29, SrTmp (T. aestivum L.), Sr2, Sr13, Sr14 (T. turgidum L.), Sr22, Sr35 (T. monococcum L.), Sr37 (T. timopheevii Zhuk.), Sr32, Sr39, Sr47, (Ae. speltoides Tausch.), Sr33, Sr45 (Ae. tauschii Coss.), Sr40 (T. araraticum Jakubz.), Sr25, Sr26, Sr43 (Ag. elongatum Host.), Sr44 (Ag. intermedium Host.), Sr27 and Sr1A.1R (S. cereale L.) (http://www.fao.org/agriculture/crops/rust/ stem/stem-pathotypetracker/stem-effectivesrgenes/en). The SrSatu gene is also effective against the Ug99 race (Olivera et al., 2013).

Based on a previous analysis of the pathogen populations virulence from the Nadira and Voevoda varieties (Baranova et al., 2023b), only the Sr32 gene is effective against both populations of the fungus among the genes, the presence of which could be assumed in the studied lines. However, this gene was not identified in any of the lines. In addition to it, the Sr39 gene, identified only in the L664 line, is effective against the Tatarstan population of the pathogen. Consequently, the lines resistance to the pathogen is determined by other unstudied genes or combinations of genes

In three lines resistant to the Volga region populations of the fungus (L935, L968 and L995/1), molecular genetic analysis failed to identify known resistance genes. Two of them (lines L935 and L968 (Table 1)) carry genetic material from S. cereale. Their pedigrees include the triticale variety Satu and they have chromosomal substitution 3R(3D) according to cytogenetic analysis (Table 2, the Figure, e). They also turned out to be immune to the Ug99 race. The L968 line is also immune to yellow rust (according to the KALRO assessment), i. e. it has resistance to yellow and stem rust pathogens. The SrSatu gene is localized on chromosome 3R of rye and is closely linked to the LrSatu gene. In addition, the Sr27 gene is localized on chromosome 3R (Singh, McIntosh, 1988). According to McIntosh (1995), the Sr27 and SrSatu genes are allelic to each other and are highly effective against the stem rust pathogen populations. In earlier work, S.J. Singh and R.A. McIntosh, based on genetic analysis of F2 and F3 hybrids of stem rust-resistant varieties Satu (SrSatu) and Coorong (Sr27) with susceptible triticale varieties, showed that the resistance of each variety is determined by one dominant gene and the SrSatu and Sr27 genes are allelic or closely linked (Singh, McIntosh, 1988). This article shows that the Satu variety used in the crosses did not carry the Sr27 gene. In our studies, the response type was “1” to the population of P. graminis f. sp. tritici collected from the spring bread wheat variety Voevoda, and “2+” to the population from the variety Nadira for the Sr27 gene (Baranova et al., 2023b), while lines L935 and L968 showed the infection type of either “0” or “1” (Supplementary Material 3). On the other hand, the Sr27 gene is effective against the Ug99 race, but the lines containing it are resistant or moderately resistant (R, MR) (Jin et al., 2007); in our study, the lines were immune, the infection type was “0” (Table 2). Thus, taking into account the pedigree, as well as data from cytogenetic and phytopathological analyses, there is reason to believe that the L935 and L968 lines carry the SrSatu gene.

Resistance to the Volga region populations of the fungus in line L995/1 (Supplementary Material 3) is most likely determined by unidentified genes on chromosome 2G of T. timopheevii (chromosomal substitution 2G(2B), or translocation T2BS.2GL) (Table 2). The Pamyati Maistrenko variety is a donor of alien introgressions and resistance to the stem rust pathogen in the L995/1 line. As is known, this variety inherited chromosomal substitution 2B(2G) from the line of spring bread wheat “Saratovskaya 29 immune L10”, which has age-related resistance to the stem rust pathogen (Laikova et al., 2013). The L995/1 line showed an intermediate type of resistance to the Ug99 race (5M).

Resistance genes, the presence of which is confirmed by cytogenetic analysis (Table 2) and pedigree analysis (Table 1), were identified in lines L664, L997 and L1110. Line L664 was resistant to both populations of the fungus collected from the spring bread wheat varieties Voevoda and Nadira and highly resistant (5RMR) to the Ug99 race. The following genes have been identified in this line: Sr25 (confirmed by the presence of a translocation on 7DL from Th. ponticum, T7DS-7DL-7Ae#1L); Sr39 and Sr47, which are located on chromosome 2S of Ae. speltoides (McIntosh et al., 2013). Population of P. graminis f. sp. tritici from the Voevoda variety is virulent to the Sr39 and Sr25 genes; however, the L664 line is highly resistant, which may be determined by an additional resistance gene from Ae. speltoides – Sr47. The type of infection for lines with the Sr47 gene, infected with the Ug99 race, is “2–” (Klindworth et al., 2012), which correlates with the results of assessing the L664 line for resistance to Ug99 – 5RMR (Table 2). Thus, there is reason to conclude that L664 carries an effective combination of Sr25 + Sr39 + Sr47 genes.

It should be noted that the use of Sr25 gene sources in breeding for resistance to stem rust is traditional for breeding centers in the Volga region. In 2009, it was reported that a fungal isolate virulent to this gene had been identified in India (Jain et al., 2009). Unfortunately, this previously highly effective gene has been losing efficiency in recent years in the Volga region (Baranova et al., 2021; Baranova et al., 2023b). However, Sr25 is still effective against the Ug99 race and may be valuable for breeding in combination with other genes such as Sr31, Sr35 and Sr36, and in this case with the Sr39 and Sr47 genes. Lines with genetic material from Ae. speltoides with the Sr39 + Sr47 genes are very promising for breeding due to their effectiveness against the Ug99 race (Klindworth et al., 2012). Among the domestic varieties of Russia, the Chelyaba 75 variety, selected by the Chelyabinsk Research Institute of Agriculture with genetic material from Ae. speltoides (the Sr39 gene), stands out and has not only group resistance to leaf and stem rusts and smut (according to the originator), but also resistance to the Ug99 race (Shamanin et al., 2011).

Lines L997 and L1110 are resistant to both Volga populations of P. graminis f. sp. tritici. Genes Sr25 (T7DS-7DL- 7Ae#1L from Th. ponticum) and Sr36 (2G(2B)), or translocation T2BS.2GL were identified in those lines (Table 2, the Figure, g–i). Resistance to the stem rust pathogen in these lines is determined by the Sr25 + Sr36 genes combination and, probably, by unidentified gene(s) from T. timopheevii on chromosome 2At. This is confirmed by the immunity of these lines to the Ug99 race, as well as the immunity of the L997 line to the yellow rust pathogen.

The resistance of the L960 line to the stem rust population from the Nadira variety and its average resistance to the Ug99 race (5MR) are most likely associated with unidentified genes from T. kiharae (Table 2).

Line L971 is moderately resistant to race Ug99 (Table 2). It turned out to be heterogeneous in resistance to the population of P. graminis f. sp. tritici collected from the spring bread wheat variety Nadira, and resistant to the fungal population from the Voevoda variety (Supplementary Material 3). Since it is known that both populations are virulent to the Sr25 gene previously identified in this line, it can be assumed that it has other resistance genes, most likely localized on chromosome 2At of T. timopheevii and/or on chromosome 2G (Table 2). Moreover, the resistance gene(s) differ from the genes previously transferred from T. timopheevii, Sr36 (T2B/2G#1) and Sr40 (T2BL/2G#2S), by greater efficiency, since populations of P. graminis f. sp. tritici collected from varieties Nadira and Voevoda are virulent to them (Baranova et al., 2023b).

The resistance of the L758 line to the Ug99 race (5R) is determined by the Sr25 gene – T7DS-7DL-7Ae#1L from Th. ponticum (Table 2).

Line L657 is resistant to the stem rust population collected from the Voevoda wheat variety and to the Ug99 race (5RMR), but does not carry any of the tested Sr genes. It is possible that unidentified or unknown Sr genes that determine the resistance of this line are localized on chromosome 6A of T. dicoccum, which replaced its chromosome 6D (Table 2, the Figure, a).

Thus, as a result of phytopathological, molecular genetic and cytogenetic analyses, lines that were immune and resistant to the Volga region populations of the fungus, as well as to the Ug99 race (Table 2), with effective combinations of resistance Thus, as a result of phytopathological, molecular genetic and cytogenetic analyses, lines that were immune and resistant to the Volga region populations of the fungus, as well as to the Ug99 race (Table 2), with effective combinations of resistance

## Conclusion

Cytogenetic analysis together with identification using DNA markers of Sr genes and phytopathological evaluation of resistance to the Ug99 P. graminis f. sp. tritici race in introgressive lines of spring bread wheat made it possible to: determine the nature of alien introgressions; establish the degree of resistance to the pathogen; identify effective Sr genes. As a result, comprehensive characterization of ten introgressive lines of spring bread wheat resistant to the Ug99 race was obtained, which allows their targeted use in the breeding of spring bread wheat for resistance to the stem rust pathogen

## Conflict of interest

The authors declare no conflict of interest.
